# Turnover intentions in a call center: The role of emotional dissonance, job resources, and job satisfaction

**DOI:** 10.1371/journal.pone.0192126

**Published:** 2018-02-05

**Authors:** Margherita Zito, Federica Emanuel, Monica Molino, Claudio Giovanni Cortese, Chiara Ghislieri, Lara Colombo

**Affiliations:** Department of Psychology, University of Turin, Turin, Italy; Groupe ESC Dijon Bourgogne, FRANCE

## Abstract

**Background:**

Turnover intentions refer to employees’ intent to leave the organization and, within call centers, it can be influenced by factors such as relational variables or the perception of the quality of working life, which can be affected by emotional dissonance. This specific job demand to express emotions not felt is peculiar in call centers, and can influence job satisfaction and turnover intentions, a crucial problem among these working contexts. This study aims to detect, within the theoretical framework of the Job Demands-Resources Model, the role of emotional dissonance (job demand), and two resources, job autonomy and supervisors’ support, in the perception of job satisfaction and turnover intentions among an Italian call center.

**Method:**

The study involved 318 call center agents of an Italian Telecommunication Company. Data analysis first performed descriptive statistics through SPSS 22. A path analysis was then performed through LISREL 8.72 and tested both direct and indirect effects.

**Results:**

Results suggest the role of resources in fostering job satisfaction and in decreasing turnover intentions. Emotional dissonance reveals a negative relation with job satisfaction and a positive relation with turnover. Moreover, job satisfaction is negatively related with turnover and mediates the relationship between job resources and turnover.

**Conclusion:**

This study contributes to extend the knowledge about the variables influencing turnover intentions, a crucial problem among call centers. Moreover, the study identifies theoretical considerations and practical implications to promote well-being among call center employees. To foster job satisfaction and reduce turnover intentions, in fact, it is important to make resources available, but also to offer specific training programs to make employees and supervisors aware about the consequences of emotional dissonance.

## Introduction

For organizations, it is important to sustain employees’ well-being and limit the turnover rate. In fact, the most competent staff, that is the most productive in terms of quantity and quality, if develop a strong intention to leave could easily find another job placement. The organizational context would run the risk of a fall in results, both quantitative and qualitative, and should face high costs for the research, the integration and the training of new productive staff [1, 2]. Traditionally, the call center occupation was considered “transitory” and suitable especially for people with low skills, since it is a poorly paid job without any career opportunities. Currently, in Italy, a country characterised by a weak labour market, high job insecurity and unemployment, call center job has become an occupation in which personnel stay for long periods of time. Considering this particular situation and the importance of understanding employees’ well-being to face it, the present study aims to detect, within the theoretical framework of the Job Demands-Resources Model [3, 4], the role of a specific job demand, emotional dissonance, and of two resources, job autonomy and supervisors’ support, in the perception of job satisfaction and turnover intentions among an Italian call center.

### The call center work

A call center can be defined as a work environment in which operators have to interact with customers by phone or other computer-based technologies [5]. Call centers, nowadays used by several companies, appeared in the early 1990s and served for organizations to reduce the costs of some services by improving customer facilities [6], and extending expectations of high service quality. The types of call center activities can be identified in inbound and outbound: the first, are suggested to have a passive role [5], since the activity is generally focused on receive calls from customers who contact the call center to complain and face with problems, whereas the second, is considered to be more active, since the operator is mostly engaged in selling and telemarketing [7].

According to studies [5, 8], call center work can be considered as a sort of advanced Taylorism, in particular for the job division, the simplification of tasks, the pressure on job timing [9]. Moreover, the activity is complicated by the continuous contact with customers who ask for information, support and help and/or express aggression and anger [10], exposing the operator to considerable negative emotions and stressful experiences [11, 12]. In call centers, sometimes, a high cognitive effort is also required when employees have to provide difficult technical answers, often without appropriate information and training resources [13]. The work activity is therefore characterized by a continuous contact with customers, which requires also communication skills and efficiency [6], and by repetitive tasks. Moreover, employees’ performance is often controlled and this limits their autonomy, leading to pressure on the daily job [12, 14].

The continuous social interaction with customers requires call center operators to regulate their emotions as part of the work, for this reason also called emotional labour or emotion work [15, 16]. The emotional labour is referred to the quality of interactions between the client and the operator [5], and it is a job demand particularly occurring in call center job, since it requires to express, during the voice-to-voice interaction, the emotions not really felt, but required by the organization [17]. Call centers operators are particularly exposed to states of emotional dissonance, which is the discrepancy between expressed and felt emotions and occurs when the organization requires to express emotions not really felt in a certain situation [5, 16]. This is critical for call center employees’ well-being because suppressing negative emotions and expressing other positive moods, even requested by the organizational rules, can lead to emotional exhaustion [18, 19]. As highlighted by Bakker and colleagues [6], well-being oriented research in call centers identified the following as main characteristics of the call center job: role stress; performance monitoring and lack of control on the activity; insufficient coaching, training and supervisors’ support; emotional exhaustion at work, consequently linked to low job satisfaction.

### The job demands-resources model, job satisfaction and turnover intentions

Among the theoretical models able to understand the several aspects affecting well-being at work, the *Job Demands-Resources model* (JD-R model) [3, 4] has received much attention by scholars. Thanks to its flexibility, in fact, the model allows to take account of many possible working conditions, making it applicable to different occupations; more than other models, such as the *Demand-Control Model* [20, 21], the JD-R model has the added value to consider both positive and negative indicators of psychological well-being or discomfort [22]. The model assumes that well-being is influenced by two main categories of factors, job demands and job resources: job demands are mainly responsible for health degradation processes; job resources are mainly responsible for motivational processes.

More specifically, job demands are defined as “those physical, psychological, social, or organizational aspects of the job that require sustained physical and/or psychological (cognitive and emotional) effort or skills and are therefore associated with certain physiological and/or psychological costs” ([3], p. 312). Job demands can be both general, crossing all type of jobs, and specific, connected with the work characteristics. As for job resources, they are defined as “those physical, psychological, social, or organizational aspects of the job that are either/or: functional in achieving work goals; reduce job demands and the associated physiological and psychological costs; stimulate personal growth, learning, and development” ([3], p. 312). Resources are important for work since they can protect workers from discomfort outcomes and help individuals in improving their performance. According to the JD-R model, resources can also buffer the impact of job demands on negative outcomes undermining the quality of working life [3, 4].

Among the outcomes considered by studies adopting this theoretical model, a major role is played, for positive outcomes, by work engagement and, for negative outcomes, by burnout [3]. However, some studies have used the model to explain also life satisfaction [22] and job satisfaction [23, 24] in specific organizational contexts, such as call centers [6]. The present study is placed on continuity with these researches.

Job satisfaction refers to the extent to which employees like, or not, their job [25] and evaluate their job and the job situation positively, or not [26]. The research on the topic [27, 28] revealed two different perceptions of job satisfaction: overall satisfaction, referring to the work as a whole, and specific satisfaction, referring to individual aspects of the work (e.g. the level of remuneration).

The importance of the job satisfaction construct is linked to its consequences, at the organizational and individual level. At the organizational level, job satisfaction influences many aspects, including the intention to change job [29], the degree of absenteeism [30] and turnover [31, 32], the qualitative and quantitative individual and group performance [33], the quality of the product/service [34], the customer satisfaction [35], the propensity to implement organizational citizenship behaviors [36] or—in case of dissatisfaction—hostile behaviors [37] such as sabotage, damage, theft or voluntary waste of resources. At the individual level, studies show a positive relationship between job satisfaction and life satisfaction [38] and a negative relationship between job satisfaction, anxiety and depression [39, 40], with consequences on well-being at work.

As for the possible organizational determinants of job satisfaction, studies found that the following characteristics have a major role: the characteristics of the work itself (type of activities, variety, possibility of feedback, etc.); the characteristics of the working environment (space, tools, relationships with colleagues, style of supervisors, etc.); the characteristics of the work organization (rhythms, schedules, shifts, etc.); the management practices and the staff development adopted by the organization (communication, training, evaluation, salary, etc.) [28, 41, 42].

This study considered also turnover intentions, since employees who are dissatisfied on the job are more likely to leave than those who are satisfied [43], also in call center work [44]. Moeover, call center work represents a stressful experience [5] which produces high absenteeism and turnover intentions [18, 45, 46, 47], representing a crucial problem for organizations using call centers to manage clients’ services [6]. In fact, turnover intentions, which are related to employees’ intent to leave their organization, can be influenced by several factors such as the labor market, relational variables or employee attitudes [14, 46, 48], but also by the perception of the quality of working life [49, 50] that can be affected by emotional dissonance, mentioned as a specific job demand in call centers. According to studies, emotional dissonance is a context-specific stressor [51], that can lead to a depletion of the individuals’ energy [12, 19], and this stressful situation can influence both job satisfaction and intention to leave the job [45]. Considering the increasing intentions to leave call centers [47, 52], indeed, it is important to consider the role of job satisfaction, highlighted by studies as a key variable able to influence employees’ turnover intentions [48]. Moreover, it is important to understand what could limit the intention to leave: studies suggest that the availability of resources can enhance the employees’ identification and involvement in the organization, that is negatively related to turnover intentions [6]. Among studies, the crucial resources in the call center context are related to: developmental opportunities; the possibility to manage and control time to do the work; social supports, particularly from supervisors, linked to coaching and clear feedback [6, 48, 52].

This study considered the role of a specific job demand linked to the call center work, the emotional dissonance, and two job resources, job autonomy and supervisors’ support, in the perception of job satisfaction and turnover intentions. As previously mentioned, emotional dissonance is crucial for call center employees and, according to studies, it has consequences on job satisfaction [8, 53]. Indeed, other studies link not only the experience of negative or positive emotions to the perception of job satisfaction, but also underline the negative relation between the emotional dissonance experienced by employees and their job satisfaction [8]. Also, negative emotional experiences resulted to be associated to turnover intentions, as emotional strained workers would leave the job causing psychological discomfort [54], and affecting the quality of working life. Therefore, we formulated the following main study hypotheses:

*Hypothesis 1a*. Emotional dissonance has a negative relation with job satisfaction.

*Hypothesis 1b*. Emotional dissonance has a positive relation with turnover intentions.

Several studies suggested the importance of the quality of relationships in organization, since they can positively influence job satisfaction, working efficiency, communication, as well as ensure greater access to other resources [4, 55]. Studies have in particular highlighted the crucial role played by the relation with supervisors [3, 4, 56, 57]. This is also suggested in the light of the role of employee coaching, intended as working partnership in which the supervisor focuses on the performance, the needs, and the development of employees [58]. The perception of support is, in fact, crucial to perceive a higher quality of working life, work engagement and less exhaustion associated to work [3, 4, 22, 55], even in call centers [6, 12]. Another job resource particularly considered by studies is job autonomy, that is the degree of discretion on the work management. Job autonomy is considered crucial not only for its direct effects on different well-being at work indicators [3], but also as moderator of the relation between job demands and well-being outcomes [59]. Moreover, among call center, the possibility to have job autonomy is linked to a lower stress, to higher job satisfaction and performance and, consequently, to lower turnover intentions [60]. Thus, we formulated the following hypotheses:

*Hypothesis 2a*. Job resources (supervisors’ support and job autonomy) have a positive relation with job satisfaction.

*Hypothesis 2b*. Job resources (supervisors’ support and job autonomy) have a negative relation with turnover intentions.

Finally, the present study considered job satisfaction as a mediator between demands and resources and a negative outcome, such as turnover intentions. This point of view lies in the JD-R model theoretical framework, according to other studies that considered job satisfaction as a mediator between job demands, job resources and employees’ behaviours [61, 62]. Moreover, research on call center employees’ perceptions of their job in relation to their intention to quit were limited [44, 45, 47].

*Hypothesis 3*. Job satisfaction has a negative relation with turnover intentions.

*Hypothesis 4*. The negative relation between job resources and turnover intentions is increased by the mediation of job satisfaction.

*Hypothesis 5*. The positive relation between emotional dissonance and turnover intentions is decreased by the mediation of job satisfaction.

**Fig 1:**
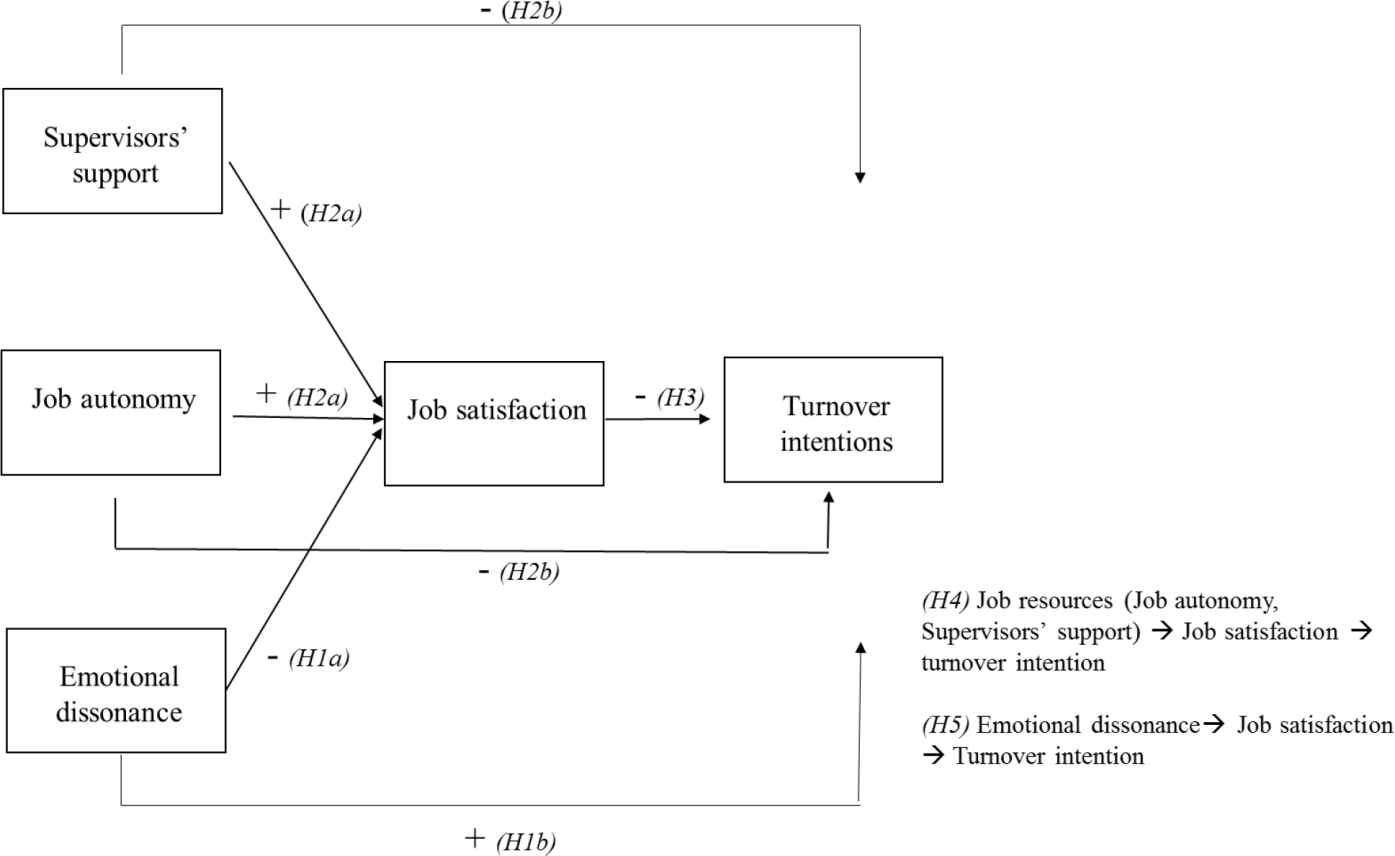
Theoretical model and expected relations.

The conceptual model and the expected relations are specifically shown in Fig 1.

## Methods

### Participants and data collection procedure

The study was conducted among a national Italian Telecommunication Company and involved a sample of inbound call center agents. Employees received an e-mail inviting them to the participation in the study explaining the aim of the research, the voluntary nature of the participation and the complete anonymity. The administration of the self-report questionnaire was on-line and the e-mail contained a link to participate in the study. The invitation to the study has been sent to 525 call center agents and 426 filled out the questionnaire (81.1% of the involved participants). After data cleaning, which excluded 108 incomplete questionnaires, the final sample comprised 318 respondents, covering the 60.6% of the call center agents involved in the research.

### Ethical statement

The research project was shared and approved by the Company Board of Directors. The research procedure has been approved by both the Scientific Committee and the multidisciplinary Technical Task Force (trade unions, health and safety managers, occupational health physicians). Since there was no medical treatment or other procedures that could cause psychological or social discomfort to participants, additional ethical approval was not required. The study was conducted according to the Helsinki Declaration [63], and data protection followed regulation of the Italian country (Legislative Decree No. 196/2003). The Company and the Department of Psychology of the University of Turin signed an agreement to ensure anonymity and confidentiality in collecting, analysing data and publishing. Participants received no reward and voluntarily participated in the research.

### Instruments

The questionnaire evaluated, through a demographic section, both personal (gender, age, marital status, having children) and professional (type of contract, time regime, seniority in the organization) characteristics of participants.

Moreover, the questionnaire assessed the following scales:

- **Turnover intentions**: 3 items of the turnover subscale of the Michigan Organizational Assessment Questionnaire [64] on a Likert scale ranging from 1 (disagree) to 4 (agree). Construct reliability (CR) was .78 and average variance extracted (AVE) was .55. The CFA indices were: *χ2* (N = 426) = 0.00, *df* = 0, *p* = 1.00; RMSEA = 0.00, the model is saturated, the fit is perfect. The Cronbach’s alpha for the present study resulted of 0.70 (M = 1.90, SD = 0.8).
- **Job satisfaction**: 3 items of the job satisfaction subscale of the OSI questionnaire by Cooper and colleagues [65] on a Likert scale ranging from 1 (very unsatisfied) to 6 (very satisfied). Construct reliability (CR) was .95 and average variance extracted (AVE) was .85. The CFA indices were: *χ2* (N = 426) = 0.00, *df* = 0, *p* = 1.00; RMSEA = 0.00, the model is saturated, the fit is perfect. The Cronbach’s alpha for the present study resulted of 0.91 (M = 3.50, SD = 1.2).
- **Job autonomy**: 6 items of the scale by Karasek and Theorell [21] on a Likert scale ranging from 1 (none) to 4 (a lot). Construct reliability (CR) was .78 and average variance extracted (AVE) was .50. The CFA indices were: *χ2* (N = 426) = 9.61, *df* = 5, *p* = ns; RMSEA = 0.05; RMR = 0.01; GFI = 0.99; AGFI = 0.99; NFI = 0.99; CFI = 0.99.The Cronbach’s alpha for the present study resulted of 0.86 (M = 2.11, SD = 1.8).
- **Emotional dissonance**: 3 items by Zapf and colleagues [17] on a Likert scale ranging from 1 (never) to 6 (always). Construct reliability (CR) was .82 and average variance extracted (AVE) was .60. The CFA indices were: *χ2* (N = 426) = 0.00, *df* = 0, *p* = 1.00; RMSEA = 0.00, the model is saturated, the fit is perfect. The Cronbach’s alpha for the present study resulted of 0.90 (M = 3.86, SD = 1.4).
- **Supervisors’ support**: 3 items by Caplan and colleagues [66] on a Likert scale ranging from 1 (disagree) to 6 (agree). Construct reliability (CR) was .78 and average variance extracted (AVE) was .55. The CFA indices were: *χ2* (N = 426) = 0.00, *df* = 0, *p* = 1.00; RMSEA = 0.00, the model is saturated, the fit is perfect. The Cronbach’s alpha for the present study resulted of 0.94 (M = 4.79, SD = 1.3).

### Data analysis

Data analysis first performed, through SPSS 22, descriptive statistics, Cronbach’s alphas (*α*), and correlations (Pearson’s *r*) between all variables.

LISREL version 8.72 was used to conduct a confirmatory factor analysis (CFA) for each scale and to examine the validity. Convergent validity (whether items can effectively reflect their corresponding factor) was examined by the Average Variance Extracted (AVE) and by the Composite Reliability (CR). All AVEs were ≥ 0.5 and CRs were ≥ 0.7, thus the scale has a good convergent validity [67, 68]. In addition, all Cronbach’s alpha values exceed 0.7, suggesting a good reliability [69]. To examine the discriminant validity (whether two factors are statistically different) we compared the square root of AVE and factor correlation coefficients: for each factor, the square root of AVE is larger than its correlation coefficients with other factors. This suggests a good discriminant validity [68, 70].

It was estimated a structural equation model with LISREL version 8.72 to asses, by a path analysis, the relation between variables and the mediation of job satisfaction between job autonomy, supervisors’ support, emotional dissonance, and turnover intentions. Relations between variables and hypotheses were specified a priori leading to the choice of a partial mediation model [71].

The goodness of the model fit was evaluated assessing the following indices, according to Kelloway’s indications [72]: the chi-square value (*χ*^2^), the *χ*^2^/df ratio (ratios between 2 and 5 indicate a good fit to the data), the RMSEA (cut-off criterion: RMSEA < 0.10 means a good fit to the data), the RMR (cut-off criterion: RMR < 0.05 indicates a good fit), the GFI (cut-off criterion: GFI > 0.9 indicates a good fit), the AGFI (cut-off criterion: AGFI > 0.9 indicates a good fit), the NFI (cut-off criterion: NFI > 0.9 indicates a good fit), the CFI (cut-off criterion: CFI > 0.9 indicates a good fit), the PNFI (cut-off criterion: PNFI between 0 and 1 with higher values indicating a good and parsimonious fit).

In order to deepen relations between all the assessed variables and to confirm the a priori tested model, alternative models were performed. In particular, six models have been estimated: one saturated, one non mediated, one fully mediated, and three partially mediated.

## Results

The demographic data show that the majority of participants are female (63.50%; N = 202), are married (74.20%; N = 236), have children (69.50%; N = 221) and have a mean age of 44 years (SD = 6.80). As for the professional characteristics of participants, most of them have a permanent contract (97.50%; N = 310), work full time (68.90%; N = 219) and, in line with the mean age, they have a job seniority of about 20 years (SD = 7.23).

As for correlations (Table 1), turnover intentions and job satisfaction show significant relations with all the assessed variables. In particular, turnover is highly and negatively correlated with job satisfaction (*r* = -.48), with job autonomy (*r* = -.34), and with supervisors’ support (*r* = -.31), whereas is positively correlated with emotional dissonance (*r* = .28). Moreover, job satisfaction is highly and positively correlated with job autonomy (*r* = .51) and supervisors’ support (*r* = .42), and negatively correlated with emotional dissonance (*r* = -.29).

**Table 1:** Means, standard deviations, Cronbach’s alphas and correlations (Pearson’s *r*).

	*M*	*SD*	1.	2.	3.	4.	5.
*1*. Turnover intentions	1.90	0.80	*(*.*70)*				
*2*. Job satisfaction	3.50	1.20	-.48**	*(*.*91)*			
*3*. Supervisors’ support	4.79	1.30	-.31**	.42**	*(*.*94)*		
*4*. Job autonomy	2.11	1.80	-.34**	.51**	.28**	*(*.*86)*	
*5*. Emotional dissonance	3.86	1.40	.28**	-.29**	-.20**	-.33**	*(*.*90)*

Note.

** *p* < .01 level. Cronbach’s alphas are on the diagonal (between brackets).

After descriptive analyses and correlations, a path analysis was performed. In order to evaluate all the possible relations and to deepen the characteristics of the assessed relations between variables in this sample, different alternative models were tested; in the end, the model 6 was chosen and confirmed as the best one. As shown in Table 2, it was performed a saturated model (model 1), a nonmediated model (model 2), a fully mediated model (model 3) and three partially mediated models. More in detail, model 4 showed the relations of resources with job satisfaction, which, in turn, has a relation with turnover; and a relation between emotional dissonance with job satisfaction and with turnover. Model 5 showed no relation between emotional dissonance and job satisfaction and turnover. Model 6 showed a relation between emotional dissonance and job satisfaction, but not a relation between emotional dissonance and turnover intentions mediated by job satisfaction. Looking at fit indices in Table 2, model 6 resulted the best one, revealing a meaning of the assessed relations.

**Table 2:** Alternative model tested with path analysis.

MODEL	*Χ*^*2*^	df	*p*	*Χ*^*2*^*/df*	RMSEA	RMR	GFI	AGFI	NFI	CFI	PNFI	Δ *Χ*^*2*^
Model 1 (Saturated)	0.00	6	1.00		0.00							
Model 2 (Nonmediated)	32.64	1	0.00	32.64	0.317	0.059	0.96	0.41	0.92	0.92	0.092	32.64
Model 3 (Fully mediated)	17.18	3	0.00	5.72	0.123	0.054	0.98	0.89	0.96	0.96	0.29	15.46
Model 4 (Partially mediated)	7.81	2	0.02	3.91	0.096	0.031	0.99	0.93	0.98	0.99	0.20	9.37
Model 5 (Partially mediated)	6.14	1	0.01	6.14	0.128	0.029	0.99	0.88	0.98	0.99	0.098	1.67
**Model 6** (Partially mediated–chosen solution)	3.56	1	0.06	3.56	0.090	0.021	1	0.93	0.99	0.99	0.099	2.58

**Fig 2:**
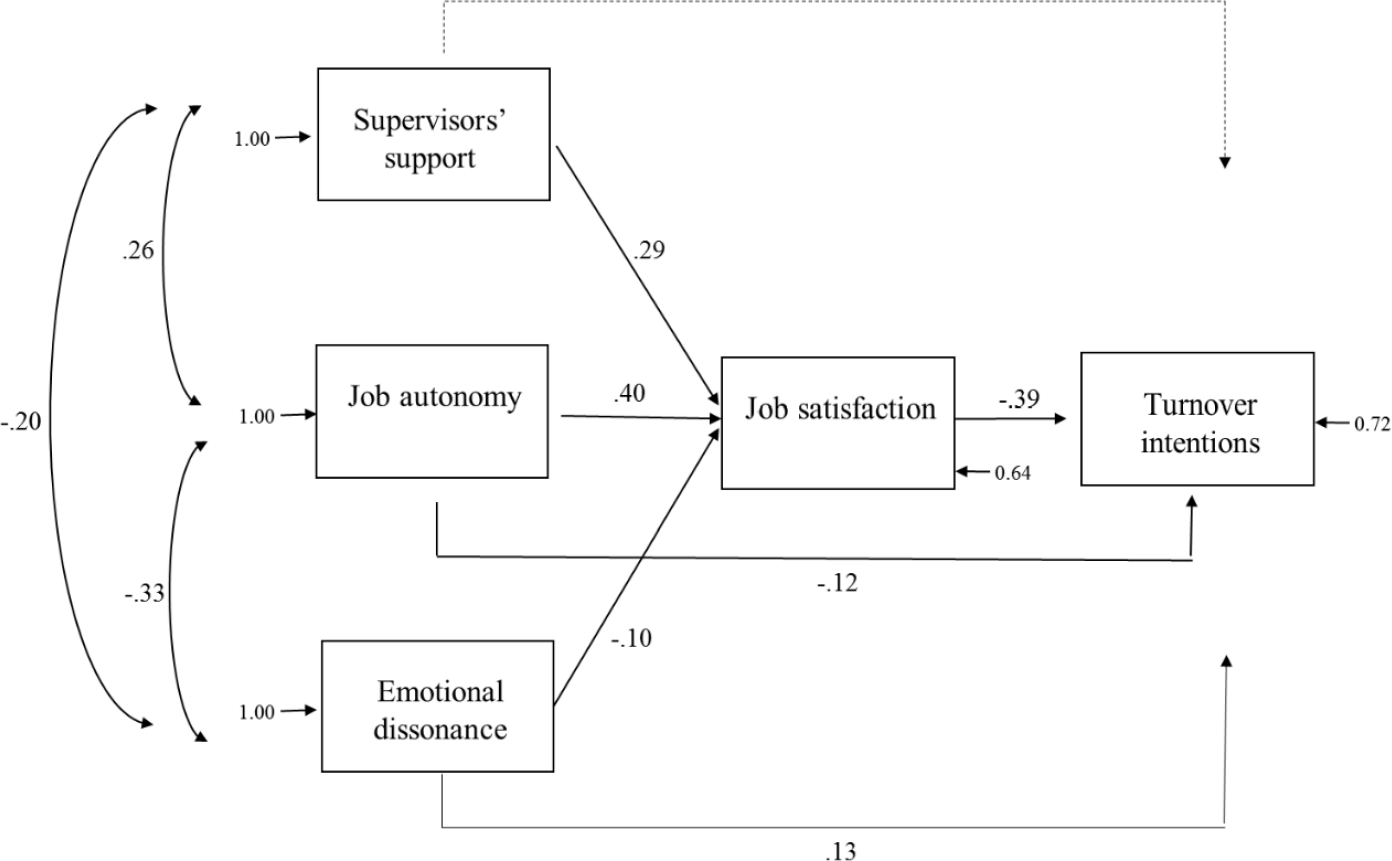
Estimated structural equations model.

This estimated selected model is shown in Fig 2 and shows very good fit indices: *χ*^2^(1) = 3.56, *p* > .005, RMSEA = 0.090; RMR = 0.021; GFI = 1; AGFI = 0.93; NFI = 0.99; CFI = 0.99. Moreover, the *χ*^2^/df ratio is 3.56, comprised in the suggested range between 2 and 5, indicating, therefore, a good fit to the data.

The model shows that the job demand, emotional dissonance, is negatively associated with job satisfaction (*β* = -.10), and positively associated with turnover intentions (*β* = .13), confirming hypotheses 1a and 1b. The model shows that, within job resources, both job autonomy (*β* = .40) and supervisors’ support (*β* = .29) are positively associated with job satisfaction, confirming hypothesis 2a. Moreover, only job autonomy is negatively associated with turnover intentions (*β* = -.12); supervisors’ support have no association with turnover intentions and this partially confirms hypothesis 2b. The direct association between job satisfaction and turnover intentions resulted negative and significant (*β* = -.39), thus confirming hypothesis 3.

As for the indirect effects performed in the model estimation, the negative association between job autonomy and turnover intentions is increased by the mediation of job satisfaction (*β* = -.16), and the negative association between supervisors’ support and turnover intentions is increased by the mediation of job satisfaction (*β* = -.12), confirming hypothesis 4. Finally, the association between emotional dissonance and turnover intentions through the mediation of job satisfaction is not significant, not confirming hypothesis 5.

## Discussion

The aim of this study was to detect the role of a job demand peculiar in call center, such as emotional dissonance, and of two job resources crucial for the quality of working life and the perception of job satisfaction and turnover intentions, such as job autonomy and supervisors’ support in an Italian call center. According to the JD-R model, in fact, the present study considered both the presence of job demands and of job resources and their importance on employee well-being [3, 4, 6]. In particular, the research considered: job satisfaction as a mediator between emotional dissonance and resources on the one hand, and turnover intentions on the other hand; whether demands and resources can predict job satisfaction and turnover; whether job satisfaction can have a role on the turnover intentions. Therefore, in the specific call center context that is subject to emotional labour and turnover, it is also important to understand what could be useful in order to limit this negative outcome and enhance employee’s well-being.

Hypotheses 1a and 1b are confirmed: expressing emotions not felt has a role on the perception of job satisfaction [8] that, in this study, seems to be decreased by emotional dissonance. Moreover, the positive association between this job demand and turnover intentions, is in line with studies suggesting that emotional dissonance could be a context stressor depleting employees’ energy, engagement and, therefore, willingness to continue carrying on a job [45, 51].

Hypothesis 2a was confirmed: in line with literature, both job autonomy and supervisors’ support are positively related to job satisfaction confirming their role as antecedents of job satisfaction and of well-being indicators in general. This type of job resources, in fact, are crucial for the quality of employees’ working life [22, 57, 59]. In particular, the perception of having support is crucial for employees’ engagement and development [3] and, thus, for their final performance. This is in line with studies suggesting a negative relationship between organizational supports and discomfort [12] and a positive relationship between these resources and employees’ well-being, underlying the role of a positive organizational climate [8, 73]. This may have consequences on employees’ intentions to leave [60]: the perceived support should have a role on turnover intentions, but even if in the correlation analysis the negative relation between supervisors’ support and turnover is confirmed, the relation in the estimated model is not significant. This could depend on the type of working context and should be deepen and developed in future studies. However, as for hypothesis 2b, as expected, job autonomy is negatively associated with turnover intentions, confirming its role as a real resource for this sample of employees, who can manage their job, and for the organization, which has more involved and motivated workers [3]. In fact, in such designed and defined job, autonomy allows employees to decide how to manage their work, in particular in answering to customers; this could permit a greater control over the relations and the negative emotions that derive from the necessity to express different emotions and behaviours. Therefore, hypothesis 2b is partially confirmed.

As expected, also hypothesis 3 is confirmed: job satisfaction is negatively related with turnover intentions, in line with studies assuming that job satisfaction can have influences on the intention to change work [29, 31, 32]. Das and colleagues [48], in fact, suggest that job satisfaction can give a measure of how people experience their quality of working life, leading them to choice if staying or leaving their job. In this sense, it could be functional to understand how fostering job satisfaction also to face and limit the possibility of turnover intentions in call center that lead organization to invest time and resources in searching and integrating new productive staff [1, 2].

The mediator role of job satisfaction between job resources and turnover supports hypothesis 4. The direct relation between supervisors’ support was not significant in the estimated model, but it is interesting that the mediation of job satisfaction is significant. The relation between supervisors’ support and turnover intentions seems to be activated by the mediation of job satisfaction which, in line with literature [3], could enhance the negative influence of the perceived support on the intention to leave the organization. This confirms that having positive and supportive organizational climate could have a role on well-being. Job satisfaction is an indicator of psychological well-being and it is less likely that satisfied workers have the intention to leave a satisfactory organization [43, 44]. Moreover, the negative relation between job autonomy and turnover is higher with the mediation of job satisfaction, suggesting that job satisfaction is a key variable on turnover dynamics [48].

Finally, hypothesis 5 has not been supported: the significant direct relation between emotional dissonance and turnover intentions, in this sample, is not decreased by the presence of job satisfaction. However, the fact that emotional dissonance has a direct significant negative relation with job satisfaction, and a direct positive relation with turnover intentions, reinforces the role of emotional dissonance as a typical job demands of call center context [12, 16], able to undermine psychological well-being and the quality of the working life.

## Conclusions and practical implications

The present study contributes, within the framework of the JD-R model, to extend the knowledge about the relations that can influence turnover intentions in call center contexts [14, 43, 44, 45, 52], leading organizations facing high costs and staff reorganization [1, 2]. Furthermore, this study contributes to deepen the role of a specific demand in call centers, emotional dissonance, that is one of the main causes of turnover among this work context [48, 52].

Useful implications for both researchers and practitioners emerged.

First of all, one important aspect refers to organizational identification and the psychological attachment to the organization. Enhancing the sense of belonging and the organizational identification can result in higher motivation, job satisfaction, organizational citizenship behaviours, and in reduced turnover intentions [36, 49, 61, 74], also among call center [44, 75]. To foster these positive dynamics is important to make employees aware about organizational job design, and to facilitate their involvement through job autonomy, which, in this study, seems to be important in particular for the potential to manage work activities and, therefore, the related emotions, and the availability of resources in general. Moreover, it is important to foster group experiences, since enhancing good dynamics between co-workers and thus creating positive relations at work could reduce the turnover intention [74].

Referring to the emotional labor, one practical action to foster awareness and involvement can be to clarify the emotional requirement during the selection process, in order to give a defined idea to individuals of what is expected [76], and also to identify the most suitable employees to perform the emotional activity [77]. In line with this, a recent study suggests that emotional job demands that are congruent with employees’ abilities are associated with job satisfaction [78].

Another implication for organizations is the development of training programs for call center operators to facilitate their emotion regulation both to cope with customer aggressions [10], and to improve emotion regulation strategies. To be aware about the consequences of emotional dissonance is a crucial aspect in order to protect employees emotional balance, improve individual strategies and reduce the negative costs associated to turnover arising from emotional strain. In line with findings of the present study, having a guide and training programs to regulate emotions could also be precious to reach job satisfaction, with positive outcome for employees’ well-being and for organizational goals.

Within this implication, also supervisors should be engaged in training programs in order to both be aware about the emotional labour, and learn and improve the support they can give to employees to overcome negative emotional situations [79]. Moreover, as emerged in this study, supervisors have a key role in supporting employees: having awareness on this topic could give the possibility to build positive organizational contexts, with good relationships and dynamics, and thus to reduce the intention to leave the organization [46, 57].

As this study shows, in fact, resources are crucial for job satisfaction and the reduction of turnover intentions. Supporting employees means to enhance the possibility to foster their well-being and their motivation [3, 4], but also their sense of belonging. Moreover, as shown by this study and suggested by several studies in the theoretical framework of the JD-R model, an important implication is to improve job autonomy and the control over activities, since studies suggest that, within call centers, autonomy is related to higher satisfaction and performance and to lower stress and turnover [60]. However, in general, organizational resources can buffer the stressful effect of job demands and can foster the individual development and abilities [22, 56]. In particular, resources as job autonomy and social support can facilitate optimal experiences at work which, in turn, can foster motivational dynamics and well-being at work [80, 81, 82].

### Limitations and future research

A first limitation of the present study is the use of a cross-sectional design of the study that does not permit to establish definite causality relationships between variables [83]. Future diary and longitudinal studies can better examine the role of emotional dissonance, resources and demands in general on job satisfaction and turnover intentions. Moreover, future longitudinal studies could overcome another limitation by detecting the level of employees’ identification with the organization, in order to verify further relations with turnover intentions and to identify adequate organizational practices.

Furthermore, this study used a self-report instrument, which may not be free from common method variance bias; this aspect could be controlled in future studies.

Another limitation refers to sample which involves only one professional group of a unique organization, not allowing the generalization of findings. Future researches could involve other groups of employees, also investigating possible differences among professional call center contexts: multi-group analysis could reveal possible differences among groups, giving a contribution in identifying best practices for employees’ well-being and reducing their intentions to leave the organization. In addition, further studies could also explore the relationship between variables and work perception, for example, in terms of perceived job insecurity, typical for the Italian labour market and for customer service jobs, as done in other professional groups [e.g. 84]. As call center job in this framework can become a long-term job, an important point to detect should be overqualification. In fact, overqualified and over-skilled workers may gain competences and skills, but not having the possibility to have a career advancement in call center. Therefore, workers may experiment job dissatisfaction or need to change job, roles or positions [85].

Moreover, future studies could detect employees’ dispositional aspects that studies indicate to be linked to job satisfaction [86], such as locus of control, the positive/negative affectivity, and emotional stability. Monitoring these variables could help the understanding of the emotional labour dynamics which characterize call centers, and that can have a detrimental effect on employees’ well-being and job satisfaction. Moreover, in line with studies detecting the relationship between negative emotional experiences at work and job satisfaction [54], these aspects could be read with organizational data on absenteeism or the use of sick leave, in order to detect and prevent potential problems related to job demands or customer aggression, and the quality of working life.

Finally, future considerations about call center contexts should contemplate the possibility for employees to adequately recover and detach during and after work. This could preserve their energy, create the conditions to live optimal experiences at work [87], which should allow them to better cope with negative emotions deriving from the emotional labor, and to improve the quality of extra-work life [88].

## Supporting information

S1 Data Matrix The data matrix with all the information for replicating the study. (SAV)

S1 Questionnaire (English version). (DOC)

S2 Questionnaire (Italian version). (DOC)
