# Supplementary material for: Turnover intentions in a call center: The role of emotional dissonance, job resources, and job satisfaction
Source: PLoS One. 2018 Feb 5;13(2):e0192126. doi: 10.1371/journal.pone.0192126 (PMC5798826; doi:10.1371/journal.pone.0192126)
Supplement: S1 Questionnaire — (DOC) [file pone.0192126.s002.doc]

**S1 Questionnaire (English version)**

**Turnover Intentions in a Call Center: the Role of Emotional Dissonance, Job Resources, and Job Satisfaction**

Margherita Zito, Federica Emanuel*, Monica Molino, Claudio Giovanni Cortese, Chiara Ghislieri, Lara Colombo

*Department of Psychology, University of Turin, Turin, Italy*

**PERSONAL AND PROFESSIONAL DETAILS**

#### d1. Gender

#### 🞎 (1) Female

#### 🞎 (2) Male

#### d2. Age: ________

d3. **Marital status**

| 🞎 (1) | Single | 🞎 (3) | Separated or Divorced |
| --- | --- | --- | --- |
| 🞎 (2) | Conjugated or Cohabitant | 🞎 (4) | Widowed |

d4. **Do you have children?**

#### 🞎 (1) Yes

#### 🞎 (2) No

**d5. What kind of employment contract do you have?**

| 🞎 (1) | Permanent contract |
| --- | --- |
| 🞎 (2) | Fixed-term contract |
| 🞎 (3) | Starter contract or Apprenticeship |

**d6. What is your work schedule?**

| 🞎 (1) | Full-time |
| --- | --- |
| 🞎 (2) | Part-time 52% - 75% |
| 🞎 (3) | Part-time 50% |

**What is your job seniority in this Company?**

d7. *Years* _____________ d7BIS. *Months* ______________

**THE RELATIONSHIP WITH THE ORGANIZATION AND WORK**

d8. **Thinking about your organization, we ask you to indicate your satisfaction with the following aspects…**

(1 - very unsatisfied to 6 - very satisfied)

|  |  | 1  *very unsatisfied* | 6  *very satisfied* |
| --- | --- | --- | --- |
| d8.1 | The work that you actually perform | ➀➁➂➃➄➅ | |
| d8.2 | The motivation that you obtain from your work | ➀➁➂➃➄➅ | |
| d8.3 | The type of work and the tasks that you have to perform | ➀➁➂➃➄➅ | |

.

d9. **Thinking about your supervisor, we ask you to answer the following questions**

# (1- disagree to 6-agree)

|  |  | Your supervisor |
| --- | --- | --- |
|  |  | 1 6  *disagree agree* |
| d9.1 | How much help does he/she offer to face a serious labor problem? | ➀ ➁ ➂ ➃ ➄ ➅ |
| d9.2 | How much he/she is available to listen to your professional problems? | ➀ ➁ ➂ ➃ ➄ ➅ |
| d9.3 | How much he/she helps you in carrying out the daily work? | ➀ ➁ ➂ ➃ ➄ ➅ |
| d9.4 | How much he/she is available to listen to your personal problems? | ➀ ➁ ➂ ➃ ➄ ➅ |

d10. In your work day, how often to...

(1 – never to 6 - always)

|  |  | 1  *never* | 6  *always* |
| --- | --- | --- | --- |
| d10.1 | Display emotions which do not correspond to inner feelings | ➀ ➁ ➂ ➃ ➄ ➅ | |
| d10.2 | Display positive emotions while feeling indifferent | ➀ ➁ ➂ ➃ ➄ ➅ | |
| d10.3 | Force yourself to show certain feelings | ➀ ➁ ➂ ➃ ➄ ➅ | |

d11. **Indicate your degree of autonomy in relation to the following situations**

(1 - none to 4 – a lot)

|  |  | 1 4  *none a lot* |
| --- | --- | --- |
| d11.1 | ... to determine the method of working yourself | ➀ ➁ ➂ ➃ |
| d11.2 | ... to choose tasks and activities to be performed | ➀ ➁ ➂ ➃ |
| d11.3 | ... to decide on the quality of your work | ➀ ➁ ➂ ➃ |
| d11.4 | ... to determine the amount of work to be done during a certain period yourself | ➀ ➁ ➂ ➃ |
| d11.5 | ... to raise or lower the pace of work yourself | ➀ ➁ ➂ ➃ |
| d11.6 | ... to pause in your work whenever you want | ➀ ➁ ➂ ➃ |

d12. **Thinking to your work, indicate your agree with the following statements**

(1 – disagree to 4 - agree)

|  |  | 1  *disagree* | 4  *agree* |
| --- | --- | --- | --- |
| d12.1 | I often think about quitting my job | ➀ ➁ ➂ ➃ | |
| d12.2 | I often read job offers | ➀ ➁ ➂ ➃ | |
| d12.3 | A job with a similar salary in another organization would be an interesting alternative to my current job | ➀ ➁ ➂ ➃ | |
