# Supplementary material for: Turnover intentions in a call center: The role of emotional dissonance, job resources, and job satisfaction
Source: PLoS One. 2018 Feb 5;13(2):e0192126. doi: 10.1371/journal.pone.0192126 (PMC5798826; doi:10.1371/journal.pone.0192126)
Supplement: S2 Questionnaire — (DOC) [file pone.0192126.s003.doc]

**S2 Questionnaire (Italian version)**

**Turnover Intentions in a Call Center: the Role of Emotional Dissonance, Job Resources, and Job Satisfaction**

Margherita Zito, Federica Emanuel*, Monica Molino, Claudio Giovanni Cortese, Chiara Ghislieri, Lara Colombo

*Department of Psychology, University of Turin, Turin, Italy*

**Sezione anagrafica**

#### d1. Sesso

#### 🞎 (1) Femminile

#### 🞎 (2) Maschile

#### d2. Età: ________

d3. **Stato civile**

| 🞎 (1) | Celibe/Nubile | 🞎 (3) | Separato/a, Divorziato/a |
| --- | --- | --- | --- |
| 🞎 (2) | Coniugato/a o Convivente | 🞎 (4) | Vedovo/a |

d4.**Ha figli?**

#### 🞎 (1) Sì

#### 🞎 (2) No

**d5.** Che tipo di contratto di lavoro ha?

| 🞎 (1) | Tempo indeterminato |
| --- | --- |
| 🞎 (2) | Tempo determinato |
| 🞎 (3) | Di inserimento/di formazione o di apprendistato |

**d6.** Qual è il suo regime orario?

| 🞎 (1) | Full-time |
| --- | --- |
| 🞎 (2) | Part-time 52% - 75% |
| 🞎 (3) | Part-time 50% |

**Qual è la sua anzianità lavorativa in questa azienda?**

d7. *Anni* _____________ d7BIS. *Mesi* ______________

**La relazione con l’organizzazione e il lavoro**

d8. **Pensando alla sua organizzazione, le chiediamo di indicare la sua soddisfazione rispetto ai seguenti aspetti**

(Scala da 1 - molto insoddisfatto a 6 - molto soddisfatto)

|  |  | 1  *Molto insoddisfatto* | 6  *Molto soddisfatto* |
| --- | --- | --- | --- |
| d8.1 | Il lavoro che lei effettivamente svolge | ➀➁➂➃➄➅ | |
| d8.2 | La motivazione che lei ricava dal suo lavoro | ➀➁➂➃➄➅ | |
| d8.3 | Il tipo di lavoro e le mansioni che lei deve svolgere | ➀➁➂➃➄➅ | |

d9. **Pensando al suo diretto superiore le chiediamo di rispondere alle seguenti domande**

# (Scala da 1 - per niente a 6 - del tutto)

|  |  | Il suo diretto superiore |
| --- | --- | --- |
|  |  | 1 6  *Per niente Del tutto* |
| d9.1 | Quanto aiuto offrono di fronte a un serio problema di lavoro? | ➀ ➁ ➂ ➃ ➄ ➅ |
| d9.2 | Quanto sono disponibili ad ascoltare i suoi problemi professionali? | ➀ ➁ ➂ ➃ ➄ ➅ |
| d9.3 | Quanto aiutano nello svolgere l’attività lavorativa quotidiana? | ➀ ➁ ➂ ➃ ➄ ➅ |
| d9.4 | Quanto sono disponibili ad ascoltare i suoi problemi personali? | ➀ ➁ ➂ ➃ ➄ ➅ |

d10. Nella sua giornata lavorativa, con quale frequenza deve…

(Scala da 1 - mai a 6 - sempre)

|  |  | 1  *Mai* | 6  *Sempre* |
| --- | --- | --- | --- |
| d10.1 | Esprimere emozioni che non corrispondono a quanto sta provando | ➀ ➁ ➂ ➃ ➄ ➅ | |
| d10.2 | Mostrare emozioni positive pur essendo indifferente | ➀ ➁ ➂ ➃ ➄ ➅ | |
| d10.3 | Sforzarsi di mostrare determinati sentimenti | ➀ ➁ ➂ ➃ ➄ ➅ | |

d11. **Indichi il suo grado di autonomia rispetto alle seguenti situazioni…**

(Scala da 1 - nessuna a 4 - molta)

|  |  | 1 4  *nessuna molta* |
| --- | --- | --- |
| d11.1 | ... nella scelta dei metodi per effettuare il suo lavoro | ➀ ➁ ➂ ➃ |
| d11.2 | ... nella scelta dei compiti o attività da svolgere | ➀ ➁ ➂ ➃ |
| d11.3 | ... nel decidere sulla qualità del suo lavoro | ➀ ➁ ➂ ➃ |
| d11.4 | ... nel decidere sulla quantità di lavoro da svolgere | ➀ ➁ ➂ ➃ |
| d11.5 | ... nel decidere sul ritmo di lavoro | ➀ ➁ ➂ ➃ |
| d11.6 | ... nel prendere una pausa dal lavoro | ➀ ➁ ➂ ➃ |

d12. **Pensando al suo lavoro, quanto è d’accordo con le seguenti affermazioni?**

(Scala da 1 - disaccordo a 4 - accordo)

|  |  | 1  *Disaccordo* | 4  *Accordo* |
| --- | --- | --- | --- |
| d12.1 | Penso frequentemente di lasciare il mio lavoro | ➀ ➁ ➂ ➃ | |
| d12.2 | Leggo spesso gli annunci di lavoro | ➀ ➁ ➂ ➃ | |
| d12.3 | Un lavoro con uno stipendio simile in un’altra organizzazione sarebbe un’alternativa interessante al mio attuale lavoro | ➀ ➁ ➂ ➃ | |
